# Hospital-at-Home care for acute heart failure: Feasibility and safety pilot

**DOI:** 10.1007/s12471-025-01949-0

**Published:** 2025-03-25

**Authors:** Jesper B. Bosman, Florine J. P. Jager, Erik A. Badings, Jan van Wijngaarden, Wouter W. Jansen Klomp

**Affiliations:** 1 Department of Cardiology, Deventer Hospital, Deventer, The Netherlands; 2 Department of Cardiology, Meander Medical Centre, Amersfoort, The Netherlands

**Keywords:** Heart failure, Home Care Services, Hospital-Based, Diuretics

## Abstract

**Introduction:**

Heart failure (HF) is a global health issue, imposing a significant burden on healthcare systems. Deventer Hospital recently introduced DZThuis, a hybrid Hospital-at-Home care model for patients with acute decompensated heart failure (ADHF). Patients receive treatment with intravenous diuretics at home when possible and in hospital when necessary. This pilot study evaluated the feasibility of DZThuis and compared outcomes with conventional in-hospital care to assess safety.

**Methods:**

This retrospective, single-centre cohort study compared 47 DZThuis patients (July 2022–November 2023) with 60 in-hospital ADHF patients admitted between August 2021 and July 2022. Kaplan-Meier curves and log-rank tests were used to analyse mortality and time to the composite endpoint of mortality or HF readmission. Secondary endpoints included total treatment duration, renal function, and complications.

**Results:**

No significant differences were found in mortality (*p* = 0.987) or time to the composite endpoint (*p* = 0.745). Treatment duration did not significantly differ (DZThuis: 11.3 ± 8.4 days vs in-hospital: 8.8 ± 4.9 days; *p* = 0.068). Complication rates were comparable. Five DZThuis patients transitioned to in-hospital care, in line with the hybrid model’s design.

**Conclusion:**

Despite a higher prevalence of comorbidities, DZThuis demonstrated outcomes comparable with traditional in-hospital care for ADHF patients and proved to be a feasible and safe model. Further long-term research in larger cohorts is needed to confirm safety and efficacy, with a particular focus on the impact of Hospital-at-Home care on quality of life and patient satisfaction.

**Supplementary Information:**

The online version of this article (10.1007/s12471-025-01949-0) contains supplementary material, which is available to authorized users.

## What’s new?

- Hospital-at-Home is a new local treatment initiative for acute decompensated heart failure patients.
- There is no difference in 3‑month all-cause mortality and hospital readmission rates between Hospital-at-Home and in-hospital patients.
- Complication rates in Hospital-at-Home and in-hospital patients are similar.

## Introduction

In the Netherlands, chronic heart failure (HF) affects approximately 1–2% of the population, with a much higher prevalence of 21% among individuals aged 85 and older [1, 2]. The Netherlands Heart Foundation estimates that 20–30% of Dutch citizens will develop HF in their lifetime [3]. Since HF patients are frequently hospitalised during episodes of decompensation, this places increasing pressure on hospital capacity and costs [4].

To address this issue, there is growing support for shifting hospital-level care to patients’ homes through so-called Hospital-at-Home (HaH) initiatives [5]. From July 2022, Deventer Hospital (DZ) introduced a version of HaH for acute decompensated heart failure (ADHF) patients: DZThuis (i.e. DZHome). Guided by the principle of ‘treatment at home when possible, in hospital when necessary,’ DZThuis patients are admitted to home care if their condition allows. There, they receive furosemide via portable continuous infusion pumps while being closely monitored. Once sufficient clinical stability is achieved, they transition back to routine outpatient care.

Previous studies have demonstrated the benefits of HaH concepts. Qaddoura et al., in a comparison of in-hospital and HaH care for ADHF patients across European settings, found no difference in all-cause mortality or time to hospital readmission [6]. However, feasibility studies on HaH initiatives in the Netherlands are scarce and do not report clinical outcomes [7].

Hence, this retrospective study aims to assess the feasibility of safely treating this patient group in a HaH setting. Additionally, we conducted an exploratory comparison with the outcomes of standard in-hospital treatment for ADHF patients.

## Methods

### DZThuis

All DZThuis patients are initially assessed at the hospital’s Emergency Heart Care to determine whether they can receive treatment at home or if they require one or more nights in hospital before completing their treatment at home. If necessary, patients can be readmitted to hospital at a later stage during the treatment.

During DZThuis treatment, the patient is visited by a cardiac care unit (CCU) nurse in the morning, and the nurse remains available 24/7 by phone if needed. During the visit, a daily history is taken, a physical examination is performed, and blood is drawn to assess renal function. Laboratory results are available in the afternoon, at which point the nurse consults the cardiologist on duty. The cardiologist then adjusts medical treatment or discharges the patient from DZThuis.

Similar to standard in-hospital care, patients are followed up by the outpatient HF clinic within two weeks of discharge. General practitioners (GPs) are not involved in the treatment unless non-cardiac conditions are suspected.

### Patients

The intervention group consists of consecutive acute decompensated heart failure (ADHF) patients admitted to DZThuis from the start of the programme in July 2022 to 1 November 2023. According to predetermined criteria, patients must have worsening heart failure requiring intravenous diuretics while remaining clinically stable enough to receive treatment at home. Therefore, patients receiving oxygen therapy or intravenous medication other than diuretics are not eligible for inclusion.

Patients with insufficient knowledge of the Dutch language, lack of internet access, or with dementia were excluded. Furthermore, patients must have adequate family support or reside in a care home to ensure daily physical care. For practical reasons, only patients living within 30 km of the hospital were eligible to participate in the programme.

If patients were accepted for DZThuis after 16.00 hrs, they spent the first night in hospital and began the programme the following morning. During the study period, up to four HF patients could participate in DZThuis simultaneously. If patients underwent multiple DZThuis treatments for separate episodes of ADHF, only their first treatment was included in the analysis.

As this is not a randomised trial, ADHF patients admitted to Deventer Hospital in the year before the start of DZThuis—between 1 August 2021 and 20 July 2022—served as the control group. Patients receiving intravenous medication other than diuretics or oxygen therapy were excluded, as these criteria would have rendered them ineligible for DZThuis.

Patients were discharged based on euvolaemia, as determined by the cardiologist’s clinical assessment in conjunction with renal function and urea levels.

### Outcomes

The primary outcomes of this study were time to all-cause mortality and time to the composite endpoint of all-cause mortality or a HF-related readmission. Follow-up was completed on 1 February 2024, ensuring a minimum of 3 months survival data for all patients. Secondary endpoints were total duration of treatment, renal function, complications (infections, delirium, or falls), changes in weight, doses of furosemide, and rates of mortality or reaching the composite endpoint at 30 days and 3 months. It was our intention to analyse quality-of-life data using the Minnesota Living with Heart Failure Questionnaire and RAND-36 questionnaire but this was not possible due to incomplete data collection and high mortality rates.

### Statistical analysis

Descriptive statistics were used to summarise baseline characteristics for both groups, and Pearson’s Chi-square tests and Fisher’s exact tests were utilised to compare them. Unpaired T‑tests were used for normally distributed data, and Mann-Whitney U tests for non-normally distributed data. Kaplan-Meier curves and log-rank tests were used to visualise and compare the primary endpoints. All statistical tests were two-tailed, with a significance level of *p* < 0.05 and were performed in IBM SPSS version 28.0.1.0.

### Ethical considerations

This study was granted approval by the local Medical Ethics Assessment Committee in Zwolle and the Review Board of the Deventer Hospital. The METC committee confirmed that the Medical Research Involving Human Subjects Act (WMO) does not apply.

## Results

### Study cohort

**Fig. 1:**
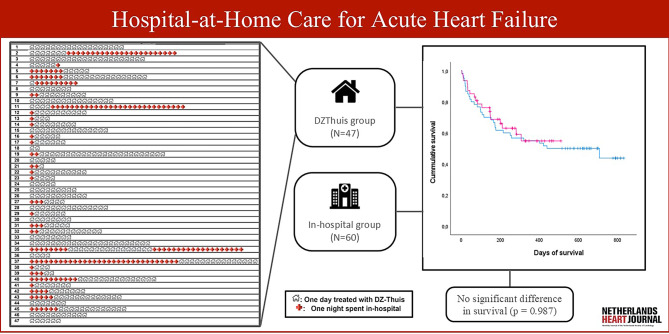
Infographic illustrating DZThuis nights spent either at-home or in-hospital

Between July 2022 and November 2023, a total of 53 treatment sessions were conducted through DZThuis, involving 48 distinct patients. One patient was found not to have ADHF during treatment and was excluded from this study, resulting in a final inclusion count of 47 patients in the DZThuis group. As a hybrid model, 10 patients spent their first night of treatment in hospital before continuing treatment at home (Fig. 1). Fourteen patients initially spent two or more nights in hospital. A total of 5 patients had to be readmitted to the hospital during their home treatment as they needed to receive additional IV medications. The primary cohort for the control group consisted of 92 patients. Exclusions were made for 4 patients found to be duplicates within the DZThuis group and 3, 3, and 22 patients who received intravenous nitroglycerin, inotropy, and antibiotics, respectively, leading to 60 patients in the in-hospital group.

### Baseline characteristics

The DZThuis group included more males compared with the in-hospital group (61.7% vs. 45.0%), although this difference was not statistically significant (*p* = 0.086) (Tab. 1). The mean age was similar in both groups (78.3 and 78.1 years; *p* = 0.922). Furthermore, no statistically significant differences were found in whether the HF was newly diagnosed (31.9% vs. 36.7%; *p* = 0.684) or if the patient had any prior HF-related admissions (27.7% vs. 31.7%) (*p* = 0.677). However, there was a significant difference between the distribution of comorbidities in the two groups, with higher rates of hypertension (*p* = 0.008), atrial fibrillation/flutter (*p* = 0.003), and history of percutaneous coronary intervention (PCI) or coronary artery bypass grafting (CABG) (*p* = 0.021) in the DZThuis group. NT-proBNP levels at presentation were not significantly different (412 vs. 478 pmol/l) (*p* = 0.963).

**Table 1:** Baseline characteristics of the in-hospital and DZ Thuis groups

	In-hospital (*n* = 60)	DZThuis (*n* = 47)	*p*-value
*Sex*			0.086
– Male	27 (45.0)	29 (61.7)	
– Female	33 (55.0)	18 (38.3)	
*Age, years, mean*	78.1 ± 9.7	78.3 ± 11.1	0.922
*Age, years, by group*			0.529
– < 65	4 (6.7)	3 (6.4)	
– 65–74	16 (26.7)	14 (29.8)	
– 75–84	23 (43.3)	12 (25.5)	
– > 85	17 (28.3)	18 (38.3)	
Systolic BP at admission, mm Hg	146 ± 28	137 ± 17	0.074
Diastolic BP at admission, mm Hg	87 ± 17	81 ± 11	0.057
Heart rate at admission, bpm	98 ± 26	93 ± 13	0.164
Weight on admission, kg	86.3 ± 21.8	87.3 ± 19.8	0.863
Height, cm	170 ± 11	173 ± 11	0.798
*Type of HF by LVEF*			0.467
– HFpEF	24 (40.0)	17 (36.2)	
– HFmrEF	10 (16.7)	5 (10.6)	
– HFrEF	25 (41.7)	25 (53.2)	
Newly diagnosed HF	22 (36.7)	15 (31.9)	0.684
Prior HF admission	19 (31.7)	13 (27.7)	0.677
*Comorbidities*			
– Hypertension	31 (51.7)	36 (76.6)	**0.008**
– Diabetes mellitus	22 (36.7)	13 (27.7)	0.324
– COPD	12 (20.0)	16 (34.0)	0.101
– Atrial fibrillation/flutter	29 (48.3)	36 (76.6)	**0.003**
– Malignancy	17 (28.3)	10 (21.3)	0.404
– Low kidney function (eGFR < 30)	15 (25.0)	8 (17.0)	0.319
– Cerebrovascular disease	10 (16.7)	6 (12.8)	0.574
– Peripheral artery disease	12 (20.0)	11 (23.4)	0.671
– Myocardial infarction	14 (23.3)	13 (27.7)	0.609
– PCI or CABG	10 (16.7)	17 (36.2)	**0.021**
– Pacemaker or ICD	8 (13.3)	8 (17.0)	0.595
NT-proBNP at admission, pmol/l	478 [242; 874]	412 [265; 829]	0.963
eGFR at admission, ml/min/1.73 m^2^	50.4 ± 23	50.1 ± 21	0.932

Variables are presented as *N* (%), mean ± SD or median (25–75th percentile)

*BP* blood pressure, *CABG* coronary artery bypass graft, *COPD* chronic obstructive pulmonary disease, *HF* heart failure, *ICD* Implantable cardioverter-defibrillator, *LVEF* left ventricular ejection fraction, *HFpEF* heart failure with a preserved ejection fraction, *HFmrEF* heart failure with a mildly reduced ejection fraction, *HFrEF* heart failure with a reduced ejection fraction, *PCI* percutaneous coronary intervention

### Primary endpoints

**Fig. 2:**
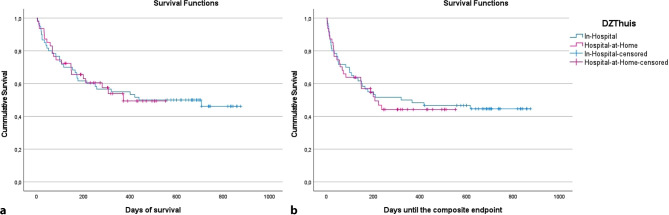
Kaplan-Meier curves of the total survival (**a**) and of reaching the composite endpoint (**b**)

Total survival in both groups was comparable (P-log-rank = 0.987) (Fig. 2a). The 30 days mortality rate was 8.5% in the DZThuis group, versus 13.3% in the in-hospital group (*p* = 0.433). For 3 months, mortality rates were 25.5 and 25.0% respectively (*p* = 0.950) (Tab. 2). The composite endpoint of death or an HF-related hospital admission was similar in both groups (P-log-rank = 0.745) (Fig. 2b). The 30-day incidence of the composite endpoint was 19.1% for the DZThuis group and 20.0% in the in-hospital group, respectively (*p* = 0.912). Rates at 3 months after discharge were 30.0% for in-hospital and 36.2% for DZThuis (*p* = 0.500) (Tab. 2).

**Table 2:** Rates of mortality and reaching the composite endpoint at 30 days and 3 months

	In-hospital (*n* = 60)	DZThuis (*n* = 47)	*P*-value
*Mortality*			
– 30 days	8 (13.3)	3 (6.4)	0.341
– 3 months	15 (25.0)	12 (25.5)	0.950
*Composite endpoint*			
– 30 days	12 (20.0)	9 (19.1)	0.912
– 3 months	18 (30.0)	17 (36.2)	0.500

In *N* (%)

### Secondary endpoints

The in-hospital group had a mean treatment duration of 8.8 ± 4.9 days, while this was 11.3 ± 8.4 for the DZThuis group (*p* = 0.068). The highest dosage of furosemide per day was higher in the DZThuis group (*p* < 0.01). The creatinine increase at discharge compared with admission was higher in the DZThuis group (*p* < 0.01). Five patients (10.6%) had to stop their home treatment and continue the treatment in-hospital. In most cases, this was due to failure of the decongestive treatment or the need to treat an infection with intravenous antibiotics. Five (8.3%) and four (8.5%) complications were registered, in the in-hospital and DZThuis group, respectively (Tab. 3).

**Table 3:** Secondary endpoints

	In-hospital (*n* = 60)	DZThuis (*n* = 47)	*p*-value
*Duration of treatment, days*	8.8 ± 4.9	11.3 ± 8.4	0.068
– Of which spent at-home	N/A	9.1 ± 5.8	
*Highest dose of furosemide, mg*			**<** **0.001**
– 40–80	23 (38.3)	3 (6.4)	
– 120–160	25 (41.7)	15 (31.9)	
– 240–360	10 (16.7)	17 (36.2)	
– > 360	2 (3.3)	12 (25.5)	
*Change in weight*			0.459
– Gained weight	4 (6.7)	3 (6.4)	
– Lost 0–5 kg	25 (41.7)	24 (51.1)	
– Lost 5–10 kg	16 (26.7)	16 (34.0)	
– Lost 10+ kg	11 (18.3)	4 (8.5)	
*Creatinine, µmol/l*			
– At admission	110 [79; 148]	116 [84; 156]	0.492
– At discharge	97 [77; 155]	124 [103; 166]	0.120
– Difference	0 [−10; 13]	12 [1; 24]	** 0.002**
*Complications*	5 (8.3)	4 (8.5)	1.0
– Infection	3 (5.0)	3 (6.4)	1.0
– Delirium	1 (1.7)	0 (0.0)	1.0
– Fall	1 (1.7)	1 (2.1)	1.0

In *N* (%), mean ± SD or median (25–75th percentile)

## Discussion

This pilot study demonstrated the feasibility of DZThuis, a hybrid HaH model. No significant differences were found in mortality, mid-term readmissions, or complications in this small group compared with a historical in-hospital patient group. This was observed despite a higher prevalence of comorbidities in the DZThuis group, consistent with findings from Qaddoura et al. in their systematic review [6]. Interestingly, a recent observational study by Sankaranarayanan et al. on a telehealth-aided virtual ward for ADHF patients reached a similar conclusion, even proposing that home treatment may be more beneficial for patients [8].

Notably, as in a recent study by Achanta et al. [9], approximately 10% of patients required transition to in-hospital care, typically for intravenous antibiotics or inotropes. We did not classify this as readmission, as in our view this is not a failure of home therapy but part of the concept of hybrid care.

One relevant but non-significant finding was a longer treatment duration in the DZThuis group. Similar trends have been reported by Mendoza et al. and Tibaldi et al., who observed prolonged total treatment durations in Hospital-at-Home (HaH) models [10, 11]. This may be attributed to the higher prevalence of comorbidities among DZThuis patients. Practical challenges that postponed adjustments in clinical management, such as laboratory results coming available in the afternoon rather than the morning, could also have played a minor role.

The average dosage of furosemide was higher in the DZThuis group, which probably also caused an increase in creatinine levels. A possible explanation is the absence of a standardised protocol for diuretic dosing and euvolaemic criteria, leaving these decisions to the discretion of the supervising cardiologist. Possibly, the more distant care made it harder to assess when a patient was completely decongested. Also, in the light of the 2022 STRONG-HF study [12], treatment regimens may have become more intense over time. Another possible, albeit speculative, explanation is lower adherence to salt and fluid restrictions in the home setting, as nurses reported observing food remnants, such as pizza boxes and soda bottles, in patients’ homes.

This study has limitations inherent to its retrospective observational design and relatively small sample size. The higher disease burden in the DZThuis group suggests potential confounding and selection bias, as the control group was formed post hoc. Non-HF related readmissions were not recorded. Additionally, the data for the in-hospital group were collected one year prior to the DZThuis group, during which time prescribing practices evolved, including greater use of SGLT‑2 inhibitors in the DZThuis group (Fig. S1 of Electronic Supplementary Material). We did not prospectively register eligible patients for home treatment who were not included in DZThuis, nor the reasons for eventual hospital treatment. This figure is estimated to be around 40%, in line with a previous research letter by Haywood et al., which estimated that 1 in 3 ADHF patients may be eligible for HaH [13]. Although our pilot study successfully demonstrated the feasibility and suggest the safety of HaH treatment, it did not thoroughly examine cost-effectiveness. Nevertheless, even without precise cost data, it was evident that the current set-up was not cost-effective. With only 53 admissions over a span of 15 months, the average number of patients receiving home treatment at any given time was 1.4, while a hospital nurse dedicated an entire shift and was on call 24/7. To address this, Deventer Hospital is actively working to make the approach more cost-efficient. Agreements have been made with home nursing care organisations to train nurses to deliver daily care under the supervision of Heart Failure physician assistants. These assistants will conduct weekly visits and consult with a cardiologist to ensure hospital-level care. To make this possible, a treatment protocol is also being developed to improve the quality and consistency of the care during admission. Future patients will continue to be monitored. Safety will be reassessed and efficacy judged with more included patients. Since maintaining a good quality of life is as important as survival for many chronically ill patients [14], assessing quality of life was initially a goal of the study. However, insufficient data were collected for this purpose. Future research should investigate the difference in quality of life between DZThuis and in-hospital care, as well as patient satisfaction with treatment.

## Conclusion

The findings from this pilot study demonstrate that it is feasible to treat ADHF patients at home with intravenous diuretics. In DZThuis, the Hospital-at-Home initiative from Deventer Hospital, there was no significant difference in total mortality and time until readmission of heart failure patients compared with the in-hospital group, despite a higher rate of patients with comorbidities in the first. Furthermore, no significant differences were seen in the rate of complications in this small pilot study with historical controls. However, larger studies are required to draw more definitive conclusions regarding safety and efficacy.

## Supplementary Information

Table showing use of medication 0–3 months after discharge
