# Supplementary material for: Hospital-at-Home care for acute heart failure: Feasibility and safety pilot
Source: Neth Heart J. 2025 Mar 25;33(5):157–62. doi: 10.1007/s12471-025-01949-0 (PMC12014988; doi:10.1007/s12471-025-01949-0)
Supplement: Supplementary file 1 — Table showing use of medication 0–3 months after discharge [file 12471_2025_1949_MOESM1_ESM.docx]

**APPENDIX #1**

Medication 0-3 months after discharge, in N (%)

|  | In-Hospital (n=60) | DZThuis (n=47) | p-value |
| --- | --- | --- | --- |
| Diuretics | 58 (96,7) | 44 (93,6) | 0,652 |
| ACE-inhibitor /ARB | 37 (61,7) | 22 (46,8) | 0,125 |
| Beta-blocker | 50 (83,3) | 31 (66,0) | 0,038 |
| SGLT-2 inhibitor | 13 (21,7) | 26 (55,3) | **<0.001** |
| Statin | 23 (38,3) | 18 (38,3) | 0,997 |
| Spironolactone | 30 (50,0) | 28 (59,6) | 0,324 |
| Platelet aggregation inhibitor | 10 (16,7) | 4 (8,5) | 0,214 |
| Vitamin K antagonist | 16 (26,7) | 7 (14,9) | 0,141 |
| DOAC | 30 (50,0) | 33 (70,2) | **0,035** |
